# Assessing how health information needs of individuals with colorectal cancer are met across the care continuum: an international cross-sectional survey

**DOI:** 10.1186/s12885-020-07539-0

**Published:** 2020-10-27

**Authors:** Hallie Dau, Abdollah Safari, Khalid Saad El Din, Helen McTaggart-Cowan, Jonathan M. Loree, Sharlene Gill, Mary A. De Vera

**Affiliations:** 1 University of British Columbia, Faculty of Pharmaceutical Sciences, 2405 Wesbrook Mall, Vancouver, BC V6T 1Z3 Canada; 2 Collaboration for Outcomes Research and Evaluation, 2405 Wesbrook Mall, Vancouver, BC V6T 1Z3 Canada; 3 BC Cancer, 855 West 12th Avenue, Vancouver, BC V5Z 1M9 Canada; 4 Faculty of Health Sciences, Simon Fraser University, Blusson Hall, 8888 University Drive, Burnaby, BC V5A 1S6 Canada; 5 Department of Medicine, Division of Medical Oncology, University of British Columbia, Faculty of Medicine, 2775 Laurel Street, 10th Floor, Vancouver, BC V5Z 1M9 Canada

**Keywords:** Colorectal cancer, Health information needs, Health survey, Health seeking behaviors, Care continuum

## Abstract

**Background:**

Studies evaluating health information needs in colorectal cancer (CRC) lack specificity in terms of study samples involving patients. We assessed how health information needs of individuals with CRC are met across the care continuum.

**Methods:**

We administered an international, online based survey. Participants were eligible for the study if they: 1) were 18 years of age or older; 2) received a diagnosis of CRC; and 3) were able to complete the online health survey in English, French, Spanish, or Mandarin. We grouped participants according to treatment status. The survey comprised sections: 1) demographic and cancer characteristics; 2) health information needs; and 3) health status and quality of life. We used multivariable regression models to identify factors associated with having health information needs met and evaluated impacts on health-related outcomes.

**Results:**

We analyzed survey responses from 1041 participants including 258 who were currently undergoing treatment and 783 who had completed treatment. Findings suggest that information needs regarding CRC treatments were largely *met*. However, we found *unmet* information needs regarding psychosocial impacts of CRC. This includes work/employment, mental health, sexual activity, and nutrition and diet. We did not identify significant predictors of having *met* health information needs, however, among participants undergoing treatment, those with colon cancer were more likely to have *met* health information needs regarding their treatments as compared to those with rectal cancer (0.125, 95% CI, 0.00 to 0.25, *p*-value = 0.051).

**Conclusions:**

Our study provides a comprehensive assessment of health information needs among individuals with CRC across the care continuum.

**Supplementary information:**

**Supplementary information** accompanies this paper at 10.1186/s12885-020-07539-0.

## Background

Colorectal cancer (CRC) is the third most common cancer and the second leading cause of cancer mortality globally [1]. The significant physical [2, 3] and psychosocial [4, 5] burden of CRC on patients underscores the need for psycho-oncology research on social, behavioural, and ethical aspects of living and dealing with CRC [6]. We previously evaluated health information *seeking behaviours* and showed similarities between individuals with young-onset CRC (yCRC, diagnosed below the age of 50 years) and average-onset CRC (aCRC, diagnosed at or above the age of 50 years) though greater reliance on digital technologies among individuals with yCRC [7], which has implications for informing age-specific resources. A relevant subsequent inquiry is on health information *needs* defined as patients’ perceptions of necessary knowledge regarding a specific health topic [8, 9]. Earlier studies evaluating health information needs in CRC lack specificity in terms of study samples involving patients with cancer with minimal representation of those with CRC [10, 11]. As well, while these studies have reported on the types of health information individuals diagnosed with CRC sought, they did not evaluate whether these information needs were met/unmet [10, 11]. More recently, in 2019 Vu et al. conducted a cross-sectional study to evaluate unmet information needs of 99 individuals with CRC after they had completed treatment [12]. Using a survey including 27 questions across six information need domains, authors reported that 74% of participants indicated at least one unmet need, with individuals with rectal cancer having more unmet need than those with colon cancer [12]. To our knowledge, there has been no specific evaluation of health information needs of individuals with CRC that consider both treatment and post-treatment phases. As such, our objectives were to: assess how health information needs of individuals with CRC are met across the care continuum, including during and following treatment; and examine factors associated with having health information needs met. As with our prior study on health information seeking behaviours in CRC [7], to guide this work, we drew from Wilson’s Second Model of Information Behaviour [13], which suggests that attainment of information needs is the necessary final component that closes the feedback loop following motivators that drive an individual to search for information and health seeking behaviours.

## Methods

### Study design and participants

This current study is nested within an international Internet-based cross-sectional study that aimed to better understand health information seeking behaviours [7] and needs among individuals with CRC. We administered the survey internationally as a reflection of growing exchange of information and support between individuals with CRC from different countries, largely due to rising popularity online communities and use of social media (e.g. Facebook groups, Instagram) [14–16]. Participants were eligible for the study if they: 1) were 18 years of age or older; 2) received a diagnosis of CRC; and 3) were able to complete the online health survey in English, French, Spanish, or Mandarin. Online, we recruited participants through Twitter, Facebook, and Instagram using our channels and those from partner CRC organizations (e.g., Colorectal Cancer Canada, COLONTOWN®, Fight Colorectal Cancer, Colorectal Cancer Alliance, and Young Adult Cancer Canada). Offline, we advertised the study using posters at waiting rooms, consultation rooms, and common spaces at two clinics providing CRC care in Vancouver, Canada. A researcher (KS) also recruited participants once a week at a local cancer centre in Vancouver, Canada, and those who consented completed the survey using a provided laptop. Finally, traditional media, which included newspaper, television, and radio interviews, were used to promote the study.

### Survey

**Fig. 1:**
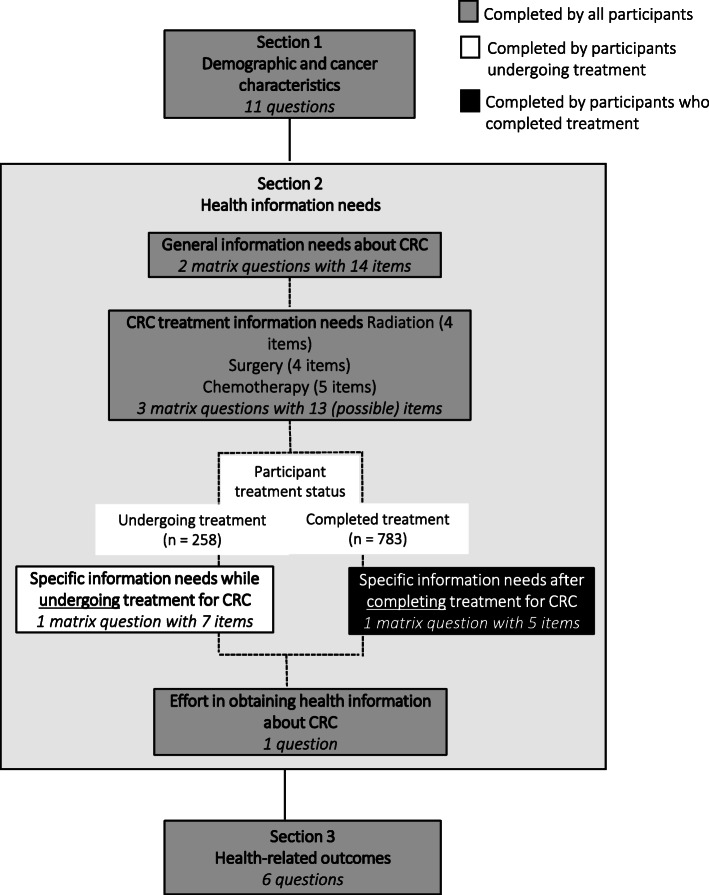
Use of computer adaptive technology facilitated administration of specific survey sections and/or items based on participants’ responses to prior questions

We administered an online health survey, which was hosted on Qualtrics, a survey platform supported by our institution and compliant with the British Columbia (BC) Freedom of Information and Protection of Privacy Act. Altogether, the survey included 12 pages online and consisted of four sections including demographic and CRC characteristics, health information needs, quality of life and health status. (Fig. 1), as well as health information seeking behaviours, which we reported in our prior study [7]. Incorporation of computer adaptive features in the survey platform facilitated administration in terms of tailoring specific items and/or sections based on participants’ responses to prior questions.

#### Demographic information and CRC characteristics

The section on demographic information and CRC characteristics comprised 12 questions on current age, sex, ethnicity, country of residence, marital status, education level, residence (e.g., urban, rural), age at CRC diagnosis, type of cancer (e.g., colon cancer, rectal cancer), cancer stage, treatment(s) received, and treatment status in terms of whether participants were currently receiving treatment for CRC or had completed treatment.

#### Health information needs

For the section on health information needs, we drew from van Mosel et al.’s 2012 scoping review of information needs across the CRC cancer care continuum [17], research team members’ clinical expertise (JL, SG), and input from patient research partners in designing questions and items (Supplementary Table 1). Questions were designed in matrix form, that is, a close-ended question that asked participants to evaluate rows of items using the same set of column choices. We explored three aspects of health information needs about CRC: **1)** *general* information needs about CRC combined two questions that captured 14 items including survival information, risk of cancer for family members, sexual activity, fertility, work/employment, and mental health; **2)** *CRC treatment* information needs included three possible questions with four (for surgery, radiation) or five (for chemotherapy) items that participants were prompted to answer (e.g., what to expect with treatment, what are treatment side effects) based on the treatment(s) they were currently receiving or had received for their CRC; and **3)** *specific* information needs according to participant treatment status with seven items for participants undergoing treatment and five for those who had completed treatment, including similar items on exercise and physical activity, nutrition and diet, and bowel activity. We applied the response format of the Cancer Survivors Unmet Needs (CaSUN) questionnaire [18]– specifically for each item, participants indicated one of the following five options: **1)** information need has been met; **2)** information need has *not* been met, need is weak; 3**)** information need has *not* been met, need is moderate; **4)** information need has *not* been met, need is strong; and **5)** information is not applicable (because it is not a need). Altogether, based on the adaptive design of our survey, the number of possible items on health information needs ranges from 25 to 34 for participants undergoing treatment and 22 to 31 for participants who have completed treatment. Finally, we also asked participants to indicate the amount of effort needed to obtain health information about CRC according to four-scales: no effort/easy to find (1), little effort/somewhat easy to find (2), moderate effort/not easy to find (3), and a lot of effort/information still not found (4).

### Analysis

Descriptive statistics, including counts and frequencies, were used to characterize participants’ sociodemographic and CRC characteristics, and group participants according to treatment status, namely those undergoing treatment and those who have competed treatment. For descriptive purposes, we applied a cut-off of having greater than 50% of *participants* indicating the response option of “information need has been met” to classify whether each item as *met* (and conversely, *unmet*). As there are no prior assessments on the extent of met health information needs among individuals of CRC to base a cut-off, we applied 50% as a conservative approach.

We then quantified met health information needs for each participant by first determining the number of specific items for each participant (e.g., number of health information need *items*). This was important since the number of health information needs items varied according to participants’ treatment status and the type of treatment modalities they received; as well some participants did not provide complete responses. Second, using the CaSUN response format, we determined whether a health information need item was *met* and then tallied the number of *met* items (e.g., number of *met* health information needs). Third, from these two values, we calculated *average met* health information needs for each participant as a relative value, ranging from 0 to 1 and expressed as a percentage, to ensure the same scale for all participants. We applied multivariable linear regression models, using the overall average number of met health information needs as the dependent variable, to determine factors associated with meeting health information needs in individuals with CRC. Factors considered included CRC characteristics (e.g., age at diagnosis, cancer type, and stage) as well as sociodemographic characteristics (e.g., location, education, marital status, and ethnicity). Separate models were computed according to treatment status. We used SAS 9.4 for the data analysis.

### Ethical approval & data sharing

This study was approved by the University of British Columbia Research Ethics Board (#H18–02540). Confidentiality of all participants was ensured by the research team. Secure firewall servers were used to store all research files and only members of the research team had access to the data. The research data are not shared.

## Results

Over the period of survey administration from November 2018 and March 2019, 1681 individuals accessed the survey and indicated their consent to participate. We excluded 556 surveys with substantial incomplete responses (e.g., only completed first online page). We further excluded 84 as these did not respond to the question on treatment status, leaving our final sample of 1041 survey respondents, 258 (24.8%) of whom were undergoing treatment and 783 (75.2%) completed treatment (Table 1). The majority of the participants were female (59.6%) and identified as white (87.7%). Most participants indicated a colon cancer diagnosis (59.2%) and were diagnosed at either Stage III (39.7%) or Stage IV (21.0%). The median duration for completion of the survey among participants was 13.6 min.

**Table 1:** Participant demographic and cancer characteristics

Characteristic	Undergoing Treatment (***n*** = 258)	Completed Treatment (***n*** = 783)	All (***n*** = 1041)	***p***-value^**a**^
**Sex**				
Female	161 (62.4)	459 (58.7)	620 (59.6)	0.2926
Male	97 (37.6)	323 (41.3)	420 (40.4)	
**Current age (years)**				
< 20	2 (0.8)	1 (0.1)	3 (0.3)	<.0001
20–29	7 (2.7)	8 (1.0)	15 (1.4)	
30–39	43 (16.7)	51 (6.5)	94 (9.0)	
40–49	72 (27.1)	110 (14.1)	182 (17.5)	
50–59	75 (29.1)	212 (27.1)	287 (27.6)	
60–69	39 (15.1)	246 (31.4)	285 (27.4)	
70–79	16 (6.2)	137 (17.5)	153 (14.7)	
> 80	4 (1.6)	18 (2.3)	22 (2.1)	
**Country**				
Canada	112 (43.4)	463 (59.1)	575 (55.2)	<.0001
USA	126 (48.4)	213 (27.2)	339 (33.0)	
UK	11 (4.3)	97 (12.4)	108 (10.4)	
Other^b^	9 (3.5)	10 (1.3)	19 (1.8)	
**Ethnicity**				
White	198 (83.9)	627 (88.9)	825 (87.7)	0.1073
Hispanic	3 (1.3)	16 (2.3)	19 (2.0)	
Black	1 (0.4)	5 (0.7)	6 (0.6)	
Asian	10 (4.2)	23 (3.3)	33 (3.5)	
Native/Aboriginal	4 (1.7)	5 (0.7)	9 (1.0)	
Middle Eastern	1 (0.4)	2 (0.3)	3 (0.3)	
Other^c^	19 (8.1)	27 (3.8)	46 (4.9)	
**Residence**				
Urban	80 (33.9)	267 (38.0)	347 (37.0)	0.4127
Suburban	97 (41.1)	257 (36.6)	354 (37.7)	
Rural	59 (25.0)	178 (25.4)	237 (25.3)	
**Education level**				
High school or less	42 (17.9)	181 (25.8)	223 (23.8)	0.0137
Postsecondary or more	193 (82.1)	521 (74.2)	714 (76.2)	
**Marital status**				
Single, never married	21 (8.8)	45 (6.4)	66 (7.0)	0.2431
Married/common-law	186 (78.2)	535 (75.9)	721 (76.5)	
Separated/divorced	24 (10.1)	92 (13.1)	116 (12.3)	
Widowed	7 (2.9)	33 (4.7)	40 (4.2)	
**Age at Diagnosis**				
yCRC	145 (56.2)	282 (36.0)	427 (41.0)	<.0001
aCRC	113 (43.8)	501 (64.0)	614 (59.0)	
**CRC Type**				
Colon	164 (63.8)	449 (57.7)	613 (59.2)	0.2159
Rectal	66 (25.7)	228 (29.3)	294 (28.4)	
Both Sites	27 (10.5)	101 (13.0)	128 (12.4)	
**CRC Stage**				
Stage 0	1 (0.4)	16 (2.1)	17 (1.6)	<.0001
Stage I	10 (3.9)	95 (12.2)	105 (10.1)	
Stage II	25 (9.7)	166 (21.3)	191 (18.4)	
Stage III	71 (27.5)	341 (43.8)	412 (39.7)	
Stage IV	136 (52.7)	82 (10.5)	218 (21.0)	
Do not know	15 (5.8)	79 (10.1)	94 (9.1)	
**Number of Treatment Modalities**				
One	54 (21.0)	214 (27.4)	268 (25.8)	0.0992
More than one	200 (77.8)	563 (72.0)	763 (73.4)	
None	3 (1.2)	5 (0.6)	8 (0.8)	
**Treatment Type**^**d,e**^				
Surgery	196 (76.0)	730 (93.2)	926 (89.0)	<.0001
Chemotherapy	235 (91.1)	558 (71.3)	793 (76.2)	<.0001
Radiation	89 (34.5)	286 (36.5)	375 (36.0)	0.5558
Other	11 (4.3)	6 (0.8)	17 (1.6)	0.0001
None	3 (0.2)	5 (0.6)	8 (0.8)	0.4030

*Abbreviations*: *yCRC* Young-onset colorectal cancer, *aCRC* Average-age onset colorectal cancer

^a^Calculated using Chi-square test

^b^Albania, Australia, China, Germany, Guinea, India, Ireland, Italy, Malaysia, Mexico, Netherlands, South Africa, and Spain

^c^Includes respondents that indicated > 1 ethnicity

^d^Multiple response answer

^e^Percentages are mutually exclusive

**Fig. 2:**
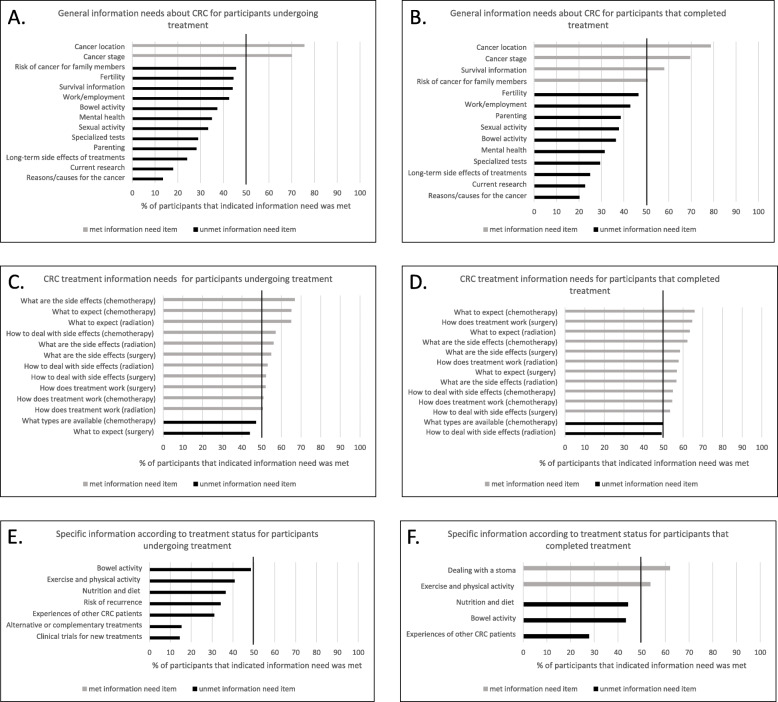
Proportion of participants indicating *met* information needs for items across three aspects of health information for CRC according to participant treatment status. **a**. Proportion of participants undergoing treatment indicating *met* general health information needs for CRC. **b**. Proportion of participants that have completed treatment indicating *met* general health information needs for CRC. **c**. Proportion of participants undergoing treatment indicating *met* CRC treatment information needs. **d**. Proportion of participants that have completed treatment indicating *met* CRC treatment information needs. **e**. Proportion of participants undergoing treatment indicating *met* specific information needs according to treatment status. **f** Proportion of participants that have completed treatment indicating *met* specific information needs according to treatment status

Figure 2 illustrates the extent that health information needs that we queried have been *met*, as represented by gray bars for items where over 50% of participants have indicated that their need for particular items have been met. For general information needs about CRC, information needs on two items (cancer location and cancer stage) were met for participants undergoing treatment (Fig. 2a) and four items (cancer location, cancer stage, survival, and risk of cancer for family members) were met for those who had completed treatment (Fig. 2b). Items that suggest areas for improvement with more than 50% of participants indicating information unmet needs include those related to CRC and associated treatments such as bowel activity and long-term side effects of treatments as well as psychosocial impacts of CRC including on work/employment, mental health, and sexual activity.

With respect to CRC treatment information needs, the majority of items were *met* for both participants undergoing treatment (11 out of 13 items *met,* Fig. 2c) and those who had completed treatment (11 out of 13 items *met,* Fig. 2d). For specific information needs according to treatment status – participants undergoing treatment had unmet needs across all seven items on 1) alternative or complementary treatments, 2) clinical trials for new treatments, 3) risk of recurrence, 4) exercise and physical activity, 5) nutrition and diet, 6) bowel activity, and 7) experiences of other CRC patients (Fig. 2e). Participants who had completed treatment indicated unmet needs for three items, namely 1) nutrition and diet, 2) bowel activity, and 3) experiences of other CRC patients (Fig. 2f).

Finally, when asked about the effort needed to find health information about CRC, 38.4% of participants undergoing treatment and 32.3% of those who had completed treatment indicated that information was “not easy to find/information still not found”.

The *average met* health information needs was 49% overall, 44% for participants undergoing treatment and 51% for those who had completed treatment. Table 2 shows the estimated coefficients of the linear regression models with the average number of health information needs as the dependent variable, according to participant treatment status and across three aspects of health information on CRC. Among participants undergoing treatment, CRC treatment information needs (Table 2b) were met to a greater extent among those with colon cancer as compared to those with rectal cancer (estimate, 0.125; 95% confidence interval [CI], 0.00 to 0.25). Findings also suggest that with respect to specific information needs according to treatment status (Table 2c), among those who completed treatment, participants with aCRC had an apparent higher average of information needs met as compared to those with yCRC (estimate, 0.063; 95% CI, 0.00 to 0.13).

**Table 2:** Regression coefficients estimates for linear regression models showing factors associated with having health information needs *met* among participants CRC according to treatment status across specific types of information

	A. General information needs about CRC				B. CRC treatment information needs				C. Specific information needs according to treatment status			
	Undergoing treatment		Completed treatment		Undergoing treatment		Completed treatment		Undergoing treatment		Completed treatment	
	Estimate (95% confidence interval)	*p*-value	Estimate (95% confidence interval)	*p*-value	Estimate (95% confidence interval)	*p*-value	Estimate (95% confidence interval)	*p*-value	Estimate (95% confidence interval)	*p*-value	Estimate (95% confidence interval)	*p*-value
Intercept	0.372 (0.25, 0.49)	<.0001	0.399 (0.31, 0.49)	<.0001	0.481 (0.31, 0.65)	<.0001	0.584 (0.45, 0.71)	<.0001	0.211 (0.08, 0.34)	0.002	0.398 (0.27, 0.52)	<.0001
**Age at Diagnosis**												
yCRC (ref)												
aCRC	0.049 (−0.03, 0.13)	0.225	0.009 (− 0.04, 0.06)	0.710	0.084 (− 0.03, 0.20)	0.143	0.004 (− 0.06, 0.07)	0.904	0.060 (− 0.03, 0.14)	0.168	0.063 (0.00, 0.13)	0.058
**Cancer Type**												
Rectal (ref)												
Colon	0.068 (− 0.02, 0.16)	0.132	0.021 (− 0.03, 0.07)	0.437	0.125 (0.00, 0.25)	0.051	0.009 (−0.06, 0.08)	0.810	0.092 (0.00, 0.19)	0.060	0.020 (− 0.05, 0.09)	0.558
Both sites	0.009 (− 0.13, 0.15)	0.902	−0.019 (− 0.09, 0.06)	0.622	0.052 (− 0.15, 0.25)	0.609	− 0.026 (− 0.13, 0.08)	0.616	0.032 (− 0.12, 0.18)	0.683	0.042 (− 0.06, 0.14)	0.395
**Location**												
Urban (ref)												
Rural	−0.068 (− 0.17, 0.03)	0.193	0.015 (− 0.04, 0.07)	0.611	−0.021 (− 0.17, 0.12)	0.771	0.004 (− 0.08, 0.09)	0.916	− 0.033 (− 0.14, 0.08)	0.562	− 0.008 (− 0.09, 0.07)	0.847
Suburban	− 0.008 (− 0.10, 0.09)	0.868	0.012 (− 0.04, 0.07)	0.654	0.036 (− 0.09, 0.17)	0.585	0.028 (− 0.05, 0.10)	0.457	0.019 (− 0.08, 0.12)	0.706	0.012 (− 0.06, 0.08)	0.749
**Education**												
College or more (ref)												
Highschool or Less	0.012 (−0.09, 0.11)	0.808	0.022 (− 0.03, 0.08)	0.410	0.014 (− 0.13, 0.15)	0.848	0.036 (− 0.04, 0.11)	0.337	0.008 (− 0.10, 0.11)	0.888	0.049 (− 0.02, 0.12)	0.169
**Marital Status**												
Common law/Married (ref)												
Separated/Divorced	0.045 (−0.08, 0.17)	0.481	− 0.023 (− 0.09, 0.05)	0.525	0.038 (− 0.14, 0.22)	0.676	− 0.073 (− 0.17, 0.02)	0.144	0.083 (− 0.05, 0.22)	0.228	− 0.053 (− 0.15, 0.04)	0.263
Single, never been married	−0.008 (− 0.16, 0.14)	0.918	0.010 (− 0.09, 0.11)	0.833	− 0.020 (− 0.22, 0.18)	0.845	− 0.030 (− 0.16, 0.10)	0.663	− 0.042 (− 0.20, 0.11)	0.594	0.077 (− 0.05, 0.20)	0.232
Widowed	0.045 (− 0.20, 0.29)	0.716	−0.040 (− 0.15, 0.07)	0.464	− 0.165 (− 0.50, 0.17)	0.334	− 0.121 (− 0.27, 0.03)	0.113	− 0.067 (− 0.33, 0.20)	0.620	− 0.040 (− 0.18, 0.10)	0.576
**Cancer Stage**												
Stage IV (ref)												
Stage 0			0.132 (−0.05, 0.31)	0.150			− 0.098 (− 0.34, 0.14)	0.424			0.004 (− 0.23, 0.24)	0.973
Stage I	−0.036 (− 0.24, 0.17)	0.726	0.034 (− 0.06, 0.13)	0.473	− 0.028 (− 0.31, 0.25)	0.844	0.041 (− 0.09, 0.17)	0.541	0.040 (− 0.18, 0.26)	0.721	0.006 (− 0.12, 0.13)	0.922
Stage II	0.058 (− 0.07, 0.18)	0.369	0.049 (− 0.04, 0.13)	0.254	0.146 (− 0.03, 0.32)	0.103	0.003 (− 0.12, 0.12)	0.964	0.087 (− 0.05, 0.22)	0.211	0.012 (− 0.10, 0.12)	0.839
Stage III	−0.045 (− 0.13, 0.04)	0.313	0.045 (− 0.03, 0.12)	0.255	− 0.107 (− 0.23, 0.02)	0.097	0.016 (− 0.09, 0.13)	0.772	0.022 (− 0.07, 0.12)	0.651	− 0.002 (− 0.11, 0.10)	0.970
Do not know	− 0.012 (− 0.19, 0.17)	0.892	0.071 (− 0.03, 0.17)	0.162	0.083 (− 0.18, 0.35)	0.539	0.010 (− 0.13, 0.15)	0.892	0.092 (− 0.10, 0.29)	0.347	0.120 (− 0.01, 0.25)	0.075
**Ethnicity**												
White (ref)												
Hispanic	−0.035 (−0.36, 0.29)	0.834	0.032 (−0.12, 0.19)	0.689	−0.069 (− 0.61, 0.47)	0.802	0.056 (− 0.16, 0.27)	0.605	0.046 (− 0.31, 0.40)	0.796	0.140 (− 0.07, 0.35)	0.190
Black	−0.328 (− 0.91, 0.25)	0.269	− 0.186 (− 0.46, 0.08)	0.175	−0.406 (−1.20, 0.38)	0.312	0.161 (− 0.20, 0.52)	0.379	− 0.223 (− 0.85, 0.41)	0.487	0.042 (− 0.31, 0.40)	0.815
Asian	0.135 (− 0.06, 0.33)	0.166	− 0.025 (− 0.16, 0.11)	0.709	0.132 (− 0.14, 0.40)	0.341	−0.071 (− 0.25, 0.11)	0.431	0.375 (0.17, 0.58)	0.000	−0.108 (− 0.28, 0.07)	0.225
Native/Aboriginal	−0.059 (− 0.35, 0.23)	0.687	−0.140 (− 0.41, 0.13)	0.300	−0.047 (− 0.50, 0.41)	0.838	−0.285 (− 0.64, 0.07)	0.113	−0.259 (− 0.57, 0.05)	0.103	−0.075 (− 0.42, 0.27)	0.674
Middle Eastern	−0.226 (− 0.79, 0.34)	0.429	−0.038 (− 0.46, 0.39)	0.859	0.194 (− 0.57, 0.95)	0.616	−0.065 (− 0.63, 0.50)	0.822	−0.017 (− 0.63, 0.59)	0.955	−0.030 (− 0.58, 0.52)	0.916
Other	−0.037 (− 0.18, 0.11)	0.617	−0.019 (− 0.14, 0.10)	0.762	−0.034 (− 0.26, 0.19)	0.766	0.043 (− 0.12, 0.21)	0.605	0.011 (− 0.15, 0.17)	0.895	0.086 (− 0.07, 0.25)	0.295

*Abbreviations*: *yCRC* Young-onset colorectal cancer, *aCRC* Average-age onset colorectal cancer

## Discussion

We administered an online survey and comprehensively assessed health information needs of individuals with CRC across the care continuum. Unique to our study, we quantified the extent that needs were met across various aspects - *general* information needs, *CRC treatment* information needs, and *specific* information needs according to participant treatment status. Our findings show that *CRC treatment* information needs were largely met for both participant groups. However, our findings of few *met* items for *general* information needs and treatment-status *specific* information needs suggest areas for improvement including *unmet* needs regarding items such as bowel activity, long-term side effects of treatments, work/employment, mental health, sexual activity, nutrition and diet, and experiences of other individuals with CRC. Our findings on the *average* number of *met* health information needs for participants as 49% overall and 44% for those undergoing treatment and 51% for those who had completed treatment suggest the need for greater emphasis on helping meet information needs for patients undergoing active treatment for CRC. This is particularly important given that the prior research on health information needs in CRC have largely focused on the period following treatment [12, 19]. Further multivariable analyses also suggest that health information needs, particularly regarding treatments, were met to a greater extent among those with colon cancer as compared to those with rectal cancer (estimate, 0.125; 95% CI, 0.00 to 0.25). As well, among those who completed treatment, participants with aCRC had an apparent higher average of information needs met as compared to those with yCRC (estimate, 0.063; 95% CI, 0.00 to 0.13).

Although the concept of heath information needs and how they are met is recognized as a key coping and adjustment strategy in response to illness, [20] this has not been extensively studied in CRC, with most earlier studies including samples of patients with cancer with very little representation from those with CRC [10, 11]. Recently in 2019, Vu et al. published a cross-sectional study of the information needs of individuals with CRC *after* treatment completion [12]. They found that those diagnosed with rectal cancer were significantly more likely to report unmet needs when compared to colon cancer [12]. Our study is unique in that it examines individuals with CRC both undergoing and those that have completed treatment and our results add to Vu et al.’s findings by showing that during treatment, health information needs were met to a greater extent among those with colon cancer as compared to those with rectal cancer (estimate, 0.125; 95% CI, 0.00 to 0.25) using a substantially larger sample. The consistency between the two papers reflects differences between rectal and colon cancer [21] with individuals with rectal cancer undergoing more involved treatments and experiencing more side effects than those with colon cancer [22], which would have implications for health information needs.

Indeed, our comprehensive evaluation of health information needs in CRC in terms of describing how these are *met* across the continuum of care as well as quantifying the extent that heath information needs for individual participants has identified both areas of strengths and needs. Indeed, our findings on the *average* number of *met* health information needs for participants as 44% for those undergoing treatment and 51% for those who had completed treatment suggest the need for greater emphasis on helping meet information needs for patients undergoing active treatment for CRC. Assessing reasons was beyond the scope of our study, however, it is possible that the emotional and psychological effects of coping with a serious diagnosis like CRC may impact how individuals process information they receive [23]. This may be particularly relevant for information regarding treatment especially if individuals are making decisions between options, which may then translate into perceptions of unmet information needs. Notably, among those undergoing treatment, two items that represented *unmet* needs, “what types of chemotherapy are available” and “what to expect with surgery” may suggest areas where CRC patients may be better supported as they are provided with information on these treatment modalities.

Participants undergoing treatment and who have completed treatment largely shared similarities in *unmet* general information needs which we were similarly queried in both groups. We note that participants who had completed treatment indicated *unmet* need for information on survival and risk of cancer for family members, which understandably reflects concerns at this stage. However, another key finding to highlight is the substantial *unmet* needs on aspects of life or activities of daily living affected by CRC including - bowel activity, long-term side effects of treatments, work/employment, mental health, sexual activity, nutrition and diet, and experiences of other individuals with CRC – which were similarly indicated by participants undergoing treatment and who had completed treatment. This suggests need for informational support for the psychosocial effects of a CRC diagnosis as well as long-term implications. In our prior, related study on the health seeking behaviors of individuals with CRC, we found that healthcare providers and the Internet were the most common sources individuals used to search for information [7]. We did not query which source they sought for which type of health information, however, it is likely that individuals with CRC may be using the Internet to search for health information not provided by their healthcare providers. Indeed, in a prior qualitative study of experiences of individuals with yCRC, participants shared that they often conducted their own independent searches on the Internet for health information on CRC, particularly on topics such as sexual activity, reproductive health, mental health, and work/employment which may not be routinely discussed during healthcare appointments, as well as connect with other patients with CRC in online communities [14]. However, although the Internet has been shown to be beneficial source of information for cancer patients, [14, 24–27] it may also lead to misinformation [28, 29]. In 2004, Al-Bahrani & Plusa assessed the quality of websites providing information to individuals with CRC and found that it can be difficult to distinguish accurate and clear information [29]. Growing collaborations between healthcare providers, researchers, and patient organizations, along with shared interests in improving care and outcomes of individuals diagnosed with CRC, represent an opportunity for the co-development and co-translation of materials and resources addressing *unmet* health information needs, particularly on psychosocial impacts of CRC.

### Study limitations

It is important to discuss strengths and limitations of our study. Working with patient research partners from partner organizations lend strength to the study, particularly with having patient feedback through survey development and pre-testing prior to the study launch. Our survey, particularly the section on health information needs was informed from a prior scoping review of information needs across the CRC cancer care continuum [17], yielding substantial coverage of items related to various aspects of health information. Nonetheless, we recognize that this is not an exhaustive list and that participants could only provide input on items that we queried and that are likely other health information need items related to CRC that we did not cover. Applying a cut-off of having greater than 50% of participants indicating the response option of “information need has been met” to classify whether each item as *met* (and conversely, *unmet*) may be considered a low threshold. However, as assessments on the extent of met health information needs have not been conducted in prior studies, our current study may serve as a foundation for evaluations of other thresholds. Nonetheless, given the descriptive nature of our assessment and reporting (Fig. 2), readily facilitates interpretation of applying other cut-offs (e.g., 60, 70%, etc) to define items as *met* (*unmet*). Gathering data through surveys may also limit the accuracy of self-reported cancer diagnosis and treatment. However, prior studies comparing self-reported cancer diagnosis with information from cancer registries have shown good sensitivity and specificity [30, 31]. The distribution of the survey online allowed us to collect an international sample of individuals with CRC. However, an online survey may have led to non-coverage bias due to convenience sampling. The participants held the decision to complete the survey and we have no knowledge about those who did not access the survey in the first place or accessed the survey and indicated their consent but dropped out before completing sections of the survey that would allow us to characterize them [32]. Given that we largely recruited participants online through social media channels, we do not have a “denominator” that is, a number that would equate to “surveys sent/mailed” as in traditional recruitment methods (e.g. using mailed surveys) and as such, are unable to determine a response rate [33]. In addition, as the survey was online, our survey was limited to individuals who have Internet access. We attempted to mitigate this using offline recruitment using television and newspaper media to promote the study, however we recognize that those reached through these offline methods were still required to access the survey online. As well, although we utilized multiple recruitment methods, capture of responses using the same platform did not allow us to distinguish how participants were recruited, nor did we ask how participants heard about the study.

We must also discuss the application of our study findings as despite our efforts to sample a diverse population, including translating the survey into other languages, our participants largely identified as White (87.8%) and had at least postsecondary education (75.9%). While we did not collect information on annual income, our study sample largely having at least postsecondary education may also suggest higher socioeconomic status. As well, the Internet-based nature of our study likely resulted in participants that represent patients who are more likely to be engaged with their care and access the Internet for information and/or support for CRC. Taken together, this largely homogeneous study population may not accurately characterize the demographic distribution of individuals with CRC. Taking this into account, it is even more striking to observe substantial *unmet* information needs, particularly regarding aspects of life or daily living activities affected by CRC (e.g., bowel activity, long-term side effects of treatments, work/employment, mental health, sexual activity, nutrition and diet, and experiences of other individuals with CRC) in this study population, who have access to resources (e.g., the Internet) and may already be quite engaged. This speaks to potential larger information gaps for under-represented and/or disadvantaged populations of individuals with CRC.

### Clinical implications

Our study highlights the similarities and differences in information needs between individuals with CRC undergoing and completed treatment. Our results have implications for informing healthcare providers to support individuals with CRC across the care continuum.

## Conclusion

Altogether, using an international survey, our study provides a comprehensive assessment of health information needs among individuals with CRC across the care continuum. Identified areas of strength include *met* information needs particularly regarding CRC treatments. Nonetheless, we also identified areas for improvement including consideration of specific information needs of individuals with rectal cancer as well as health information needs on psychosocial impacts of CRC. Finally, to address disparities in CRC, it is important to address health information needs of under-represented populations and the importance of future work recruiting more diverse study populations facilitated by possible collaborations between researchers, healthcare providers, CRC patient organizations, and community organizations.

## Supplementary information

**Additional file 1: Supplementary Table 1.** Survey items on health information needs according to participant treatment status. ^a^*For participants undergoing treatment, possible number of survey items is 25 (minimum) to 34 (maximum)*. ^*b*^*For participants who have completed treatment, possible number of survey items is 22 (minimum) to 31 (maximum)*. ^*c*^*In subsection 1, all items are administered to all participants*. ^*d*^*In subsection 2, participants respond to items based on prior question on type(s) of CRC treatment received*. ^*e*^*In subsection 3, items are administered according to participant treatment status (undergoing treatment* vs. *completed treatment).*

## Data Availability

The data that support the findings of this study are not publicly available due to them containing information that could compromise research participant privacy/consent.

## Abbreviations

BC: British Columbia
CaSUN: Cancer Survivors Unmet Needs
CRC: Colorectal cancer
aCRC: Average-age onset colorectal cancer
yCRC: Young-onset colorectal cancer
CI: Confidence intervals
UK: United Kingdom
USA: United States of America
