# Supplementary material for: Assessing how health information needs of individuals with colorectal cancer are met across the care continuum: an international cross-sectional survey
Source: BMC Cancer. 2020 Oct 27;20:1031. doi: 10.1186/s12885-020-07539-0 (PMC7590465; doi:10.1186/s12885-020-07539-0)
Supplement: Supplementary file 1 — Additional file 1: Supplementary Table 1. Survey items on health information needs according to participant treatment status. aFor participants undergoing treatment, possible number of survey items is 25 (minimum) to 34 (maximum). bFor participants who have completed treatment, possible number of survey items is 22 (minimum) to 31 (maximum). cIn subsection 1, all items are administered to all participants. dIn subsection 2, participants respond to items based on prior question on type(s) of CRC treatment received. eIn subsection 3, items are administered according to participant treatment status (undergoing treatment vs. completed treatment). [file 12885_2020_7539_MOESM1_ESM.docx]

**Supplementary Table 1. Survey items on health information needs according to participant treatment status**

| **Undergoing Treatment^a^** | **Completed Treatment^b^** |
| --- | --- |
| **General information needs about CRC^c^** | |
| (1) Cancer location | (1) Cancer location |
| (2) Cancer stage | (2) Cancer stage |
| (3) Survival information | (3) Survival information |
| (4) Reasons/causes for the cancer | (4) Reasons/causes for the cancer |
| (5) Risk of cancer for family members | (5) Risk of cancer for family members |
| (6) Current research | (6) Current research |
| (7) Specialized tests (e.g. biomarkers) | (7) Specialized tests (e.g. biomarkers) |
| (8) Sexual activity | (8) Sexual activity |
| (9) Fertility | (9) Fertility |
| (10) Work/employment | (10) Work/employment |
| (11) Parenting | (11) Parenting |
| (12) Mental health | (12) Mental health |
| (13) Bowel activity | (13) Bowel activity |
| (14) Long-term side effects of treatments | (14) Long-term side effects of treatments |
| **CRC treatment information needs**^d^ | |
| **Radiation** |  |
| (1) What to expect | (1) What to expect |
| (2) What are the side effects | (2) What are the side effects |
| (3) How to deal with side effects | (3) How to deal with side effects |
| (4) How does treatment work | (4) How does treatment work |
| **Surgery** |  |
| (1) What to expect | (1) What to expect |
| (2) What are the side effects | (2) What are the side effects |
| (3) How to deal with side effects | (3) How to deal with side effects |
| (4) How does treatment work | (4) How does treatment work |
| **Chemotherapy** |  |
| (1) What to expect | (1) What to expect |
| (2) What are the side effects | (2) What are the side effects |
| (3) How to deal with side effects | (3) How to deal with side effects |
| (4) How does treatment work | (4) How does treatment work |
| (5) What types are available | (5) What types are available |
| **Specific information needs according to treatment status^e^** | |
| (1) Alternative or complementary treatments |  |
| (2) Clinical trials for new treatments |  |
| (3) Risk of recurrence |  |
| (4) Exercise and physical activity | (1) Exercise and physical activity |
| (5) Nutrition and diet | (2) Nutrition and diet |
| (6) Bowel activity | (3) Bowel activity |
| (7) Experiences of other CRC patients | (4) Experiences of other CRC patients |
|  | (5) Dealing with a stoma |

^a^*For participants undergoing treatment, possible number of survey items is 25 (minimum) to 34 (maximum)*

*^b^For participants who have completed treatment, possible number of survey items is 22 (minimum) to 31 (maximum)*

*^c^In subsection 1, all items are administered to all participants*

*^d^In subsection 2, participants respond to items based on prior question on type(s) of CRC treatment received*

*^e^In subsection 3, items are administered according to participant treatment status (undergoing treatment vs. completed treatment)*
